# Comparative clinical significance and biological roles of PFKFB family members in oral squamous cell carcinoma

**DOI:** 10.1186/s12935-023-03110-6

**Published:** 2023-11-02

**Authors:** Kai-Fang Hu, Chih-Wen Shu, Cheng-Hsin Lee, Ching-Jiunn Tseng, Yu-Hsiang Chou, Pei-Feng Liu

**Affiliations:** 1 Institute of Clinical Medicine, National Yang Ming Chiao Tung University, Taipei, 112304 Taiwan; 2 Department of Dentistry, Division of Periodontics, Kaohsiung Medical University Hospital, Kaohsiung, 80708 Taiwan; 3 Institute of BioPharmaceutical Sciences, National Sun Yat-Sen University, Kaohsiung, 80424 Taiwan; 4 Center of Excellence for Metabolic Associated Fatty Liver Disease, National Sun Yat-Sen University, Kaohsiung, Taiwan; 5 Department of Biomedical Science and Environmental Biology, Kaohsiung Medical University, Kaohsiung, 80708 Taiwan; 6 Department of Medical Education and Research, Kaohsiung Veterans General Hospital, Kaohsiung, 813414 Taiwan; 7 School of Dentistry, College of Dental Medicine, Kaohsiung Medical University, Kaohsiung, 80708 Taiwan; 8 Department of Medical Research, Kaohsiung Medical University Hospital, Kaohsiung, 80708 Taiwan; 9 Center for Cancer Research, Kaohsiung Medical University, Kaohsiung, 80708 Taiwan; 10 Institute of Biomedical Sciences, National Sun Yat-Sen University, Kaohsiung, 80424 Taiwan

**Keywords:** Phosphofructokinase-fructose bisphosphatases, Oral squamous cell carcinoma, Clinical significance, Biological roles

## Abstract

**Background:**

Cancer cells promote glycolysis, which supports rapid cell growth and proliferation. Phosphofructokinase-fructose bisphosphatases (PFKFBs), a family of bidirectional glycolytic enzymes, play key roles in the regulation of glycolysis in many types of cancer. However, their roles in oral squamous cell carcinoma (OSCC), the most common type of oral cancer, are still unknown.

**Methods:**

We compared the gene expression levels of PFKFB family members and analyzed their clinical significance in oral cancer patients, whose clinical data were obtained the Cancer Genome Atlas database. Moreover, real-time quantitative polymerase chain reaction, western blotting, assays for cell viability, cell cycle, cell migration and viability of cell spheroid were performed in scramble and PFKFB-silenced cells.

**Results:**

We discovered that PFKFB3 expression in tumor tissues was slightly higher than that in tumor adjacent normal tissues but that PFKFB4 expression was significantly higher in the tumor tissues of oral cancer patients. High PFKFB3 and PFKFB4 expression had different effects on the prognosis of oral cancer patients with different clinicopathological outcomes. Our data showed that PFKFB3 and PFKFB4 play different roles; PFKFB3 is involved in cell viability, G2/M cell cycle progression, invasion, and migration, whereas PFKFB4 is involved in the drug resistance and cancer stemness of OSCC cells. Furthermore, oral cancer patients with co-expressions of PFKFB3/cell cycle or EMT markers and PFKFB4/stemness markers had poor prognosis.

**Conclusions:**

PFKFB3 and PFKFB4 play different biological roles in OSCC cells, which implying that they might be potential prognostic biomarkers for OSCC patients with certain clinicopathological outcomes.

**Supplementary Information:**

The online version contains supplementary material available at 10.1186/s12935-023-03110-6.

## Introduction

Oral squamous cell carcinoma (OSCC), which constitutes more than 90% of oral cancers, originates in areas of the oral cavity, including the lip, tongue, and cheek [1]. The incidence of OSCC is increasing in many countries, especially in individuals aged under 45 years [2]. Although various treatments for OSCC are available, including surgery, chemotherapy, and chemoradiation, low public awareness and insufficient screening methods have resulted in a low 5-year survival rate and poor prognosis for OSCC patients [3]. Accurate diagnostic and prognostic biomarkers are thus urgently required.

Cancer cells alter their glycolytic metabolism under aerobic conditions to maintain the high energy levels required for their growth and proliferation [4]. Aerobic glycolysis (or Warburg effect) regulates the tumorigenesis and prognosis of OSCC [5]. Several anticancer drugs targeting to glucose metabolism enzyme such as glucose transferase, hexokinase, phosphofructokinase, pyruvate kinase, lactate dehydrogenase have been developed [6]. Thus, elucidating more precise metabolic enzymes regarding to glycolytic metabolism in OSCC could provide new biomarkers or therapeutic targets for OSCC patients.

Phosphofructokinase-fructose bisphosphatases (PFKFBs), a family of bidirectional glycolytic enzymes, modulate the formation and degradation of fructose-2,6-bisphosphate (F-2,6-BP), thereby regulating glycolysis [7]. PFKFBs is encoded by four genes (*PFKFB1*, *PFKFB2*, *PFKFB3*, and *PFKFB4*) in humans [8]. *PFKFB1* is found in the liver and skeletal muscles, *PFKFB2* is found in cardiac muscles, *PFKFB3* is ubiquitously expressed, and *PFKFB4* occurs mainly in the testes [8, 9]. *PFKFB1* expression has not been detected in any cancers. The expression of *PFKFB2*, *PFKFB3*, and *PFKFB4* has been observed in several types of cancers. For example, *PFKFB2* has been highly expressed in lung cancer [10], gastric cancer [11], retinoblastoma [12], osteosarcoma [13], and breast cancer [14]. The overexpression of *PFKFB3* was observed in breast cancer [15], colon cancer [16], non-small cell lung cancer (NSCLC) [17], and hepatocellular carcinoma (HCC) [18]. The overexpression of *PFKFB4* was found in breast cancer [19], triple-negative breast cancer (TNBC) [20], osteosarcoma [21], cervical cancer [22], clear-cell renal cell carcinoma [23], melanoma[24], HCC [18], glioblastoma [25], bladder cancer [26], gastric cancer [27], pancreatic cancer [28], and prostate cancer [29]. *PFKFB2* is related to the cell proliferation, invasion, and migration of lung cancer [10]. *PFKFB3* has emerged as a key oncogene in several types of cancer; it plays a considerable role in the regulation of glycolysis in cancer cells and in the proliferation and survival of cancer cells [30]. PFKFB4 promotes chemoresistance in clear-cell renal cell carcinoma [23]. The distinct activity, synthesis, distribution, and function of *PFKB1-4* were determined by different conditions or response to different physiological or pathological stimuli [8]. However, most studies only focused on investigating the role of a member of *PFKFB* family, the roles of a set of *PFKFB* family members in cancer, especially in OSCC, remain unknown.

In the study, we performed a comprehensive analysis of the expression levels and prognostic value of a set of PFKFB family members in oral cancer patients. We found that oral cancer patients with high expression of *PFKFB3* and *PFKFB4* had poor prognosis. We also investigated their biological roles in OSCC cells, which *PFKFB3* is linked to critical aspects such as cell survival, G2/M cell cycle progression, invasion, and migration, while *PFKFB4* is strongly associated with drug resistance and the acquisition of cancer stemness characteristics. The study first reports the clinical significance and biological roles of *PFKFB3* and *PFKFB4*, which could provide potential and specific biomarkers or therapeutic targets for OSCC patients.

## Materials and methods

### Cell culture

Two OSCC cell lines, namely SAS and TW2.6 cells, were grown in Dulbecco’s modified Eagle’s medium (DMEM, Gibco™, Carlsbad, CA, USA), to which 10% heat-inactivated fetal bovine serum (Biological Industries, Cromwell, CT, USA), 1% minimum essential medium nonessential amino acids, 100 U/mL penicillin, and 100 U/mL streptomycin (Invitrogen Life Technologies, Carlsbad, CA, USA) were added, then stored at 37 °C in a 5% CO_2_ atmosphere.

### Transient transfection

The cells (2 × 10^5^ cells/well, 6 wells) were transfected with 10 nM scramble siRNA or siRNA against *PFKFB3* or *PFKFB4* (Ambion, Austin, TX, USA) for 72 h by using an RNAiMAX transfection kit (Invitrogen Life Technologies, Carlsbad, CA, USA).

### Real-time quantitative polymerase chain reaction (RT-qPCR)

Total RNA was isolated from cells by using a TRIzol reagent and then reverse transcribed using SuperScriptIII RNase Reverse Transcriptase in accordance with the manufacturer’s instructions (Invitrogen Life Technologies, Carlsbad, CA, USA). The expression levels of the genes were analyzed using SYBR Green Master Mix and QuantStudio real-time polymerase chain reaction systems (Applied Biosystems, Foster City, CA, USA). *PFKFB3* primer (Forward 5′-GGGACCGACGACACGC-3′; Reverse 5′-ATCTTCTGCACTCGGCTCTG-3′), *PFKFB4* primer (Forward 5′-TCCCCACGGGAATTGACAC-3’; Reverse 5′-GGGCACACCAATCCAGTTCA-3′) *Slug* primer (Forward 5′-TGTGACAAGGAATATGTGAGCC-3’; Reverse 5′- TGAGCCCTCAGATTTGACCTG-3′), *E-cadherin* primer (Forward 5′-ATTTTTCCCTCGACACCCGAT-3′; Reverse 5′-TCCCAGGCGTAGACCAAGA-3′), *CD166* primer (Forward 5′-ACTTGACGTACCTCAGAATCTCA -3′; Reverse 5’-CATCGTCGTACTGCACACTTT -3′), *CD44* primer (Forward 5′-CTGCCGCTTTGCAGGTGTA -3′; Reverse 5′-CATTGTGGGCAAGGTGCTATT-3’), *ABCG2* primer (Forward 5′-TGAGCCTACAACTGGCTTAGA-3′; Reverse 5’-CCCTGCTTAGACATCCTTTTCAG-3′), *aldehyde dehydrogenase 1 family, member A1* (*ALDH1A1)* primer (Forward 5’-CCGTGGCGTACTATGGATGC-3′; Reverse 5′-GCAGCAGACGATCTCTTTCGAT-3′), *aldehyde dehydrogenase 1 family, member A2* (*ALDH1A2)* primer (Forward 5’-GGGTGTGTTTTGATGCAGCCT-3′; Reverse 5′-TGGTGGGGTCAAAGGGACT-3′), and *EpCAM* (Forward 5′-AATCGTCAATGCCAGTGTACTT-3′; Reverse 5′- TCTCATCGCAGTCAGGATCATAA-3′) primer were used for mRNA amplification. The internal control *GAPDH* gene was used for normalization.

### Western blotting

Following electrophoretic separation, proteins were transferred from a polyacrylamide gel onto a nitrocellulose membrane (Millipore, Billerica, MA, USA). Blocked membranes with 5% skim milk were incubated with *PFKFB3* (ab181861, Abcam, Trumpington, Cambridge, UK) or *PFKFB4* (ab137785, Abcam, Abcam, Trumpington, Cambridge, UK) antibody overnight at 4 °C then with the horseradish peroxidase-conjugated secondary antibody at room temperature for 1 h. The ECL reagent was used for chemiluminescent detection using the a Syngene GeneGnome XRQ chemiluminescence imaging system (GeneGnome XRQ, SYNGENE, Cambridge, UK).

### Cell viability

Cell viability (6 × 10^5^ cells/mL, 96 wells) was analyzed using a CellTiter-Glo luminescent cell viability assay kit (Promega, Madison, WI, USA) in accordance with the method used in our previous study [31].

### Cell cycle assay

The cells fixed by ice-cold 75% ethanol were stained with propidium iodide (50 µg/mL, Sigma-Aldrich, St. Louis, MO, USA) then analyzed with the FACScan analyzer (Becton, Dickinson and Company, Franklin Lakes, NJ, USA). The percentages of cell cycle distribution were analyzed using FlowJo software (Tree Star, Ashland, OR, USA).

### Wound-healing assay

IBIDI Culture-Inserts (IBIDI, Inc., Planegg, Bavaria, Germany) was used to analyze cell migration. The procedure for the wound-healing assay is described in our previous study [32].

### Sensitivity of cell spheroids to drug treatment

The OSCC cells (5 × 10^3^/mL) were seeded into a 96-well, round-bottom, ultra-low-attachment microplate (Corning Costar, Cambridge, MA, USA) for cell spheroid formation. The viability of cell spheroids treated or untreated with 50 μM of cisplatin (CIS; Sigma-Aldrich Corporation, St. Louis, Missouri, USA) or 25–200 nM of paclitaxel (PTX; Selleckchem, Houston, TX, USA) for 24 h was analyzed using the CellTiterGlo 3D assay (Promega, Madison, WI, USA).

### Statistical analysis

Transcriptome data on 30 tumor-adjacent normal tissues and 315 tumor tissues from oral cancer patients were downloaded from the public Cancer Genome Atlas (TCGA) database (https://cancergenome.nih.gov). All gene expression levels and survival rate were analyzed using SPSS software (version 20.0, SPSS Inc., Chicago, IL, USA). Student’s *t* test was used to compare PFKFBs between tumor-adjacent normal tissues and tumor tissues. Univariate and multivariate Cox proportional hazards models were used to analyze survival; overall survival (OS), progression-free interval survival (PFI), disease-specific survival (DSS), and disease-free interval survival (DFI) were defined using the time intervals from the TCGA database. Cumulative survival curves were estimated using the Kaplan–Meier method. A receiver operating characteristic curve was used to determine high and low expression levels of *PFKFB* family members.

## Results

### Comparison of the expression of PFKFB family members between normal tissues and tumor tissues in oral cancer patients

**Fig. 1:**
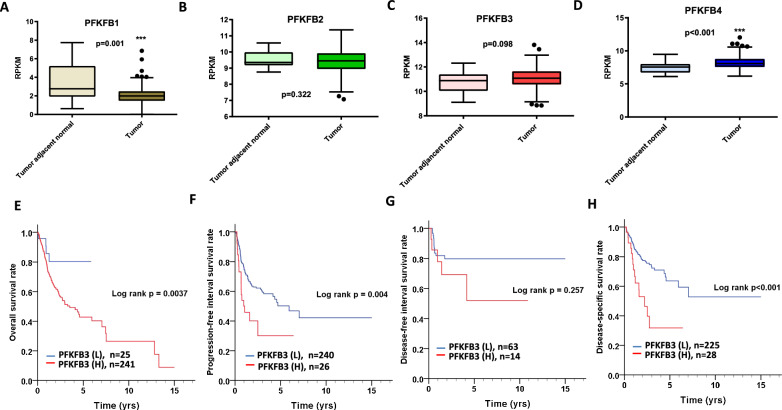
Expression and prognostic roles of PFKFB family members in oral cancer patients. Comparison of **A** *PFKFB1* **B** *PFKFB2* **C** *PFKFB3* **D** *PFKFB4* expression between 30 tumor- adjacent normal and 315 tumor tissues of oral cancer patients. The association of high and low levels of *PFKFB3* with **E** overall survival (OS), **F** progression-free interval survival (PFI), **G** disease-free interval survival (DFI) and **H** disease-specificl survival (DSS)

*PFKFB* family members differentially express in many cancer patients [8]. However, their expression levels in oral cancer patients are still unknown. After analyzing transcriptome data of oral cancer patients from TCGA database, we found that *PFKFB1* expression was lower in the tumor tissues than in the tumor-adjacent normal tissues (p = 0.001, Table 1, Fig. 1A), but *PFKFB2* expression did not differ significantly (p = 0.322; Table 1, Fig. 1B). *PFKFB3* expression was slightly higher in the tumor tissues than in the tumor-adjacent normal tissues (p = 0.098, Table 1, Fig. 1C). *PFKFB4* expression in tumor tissues was significantly higher than that in normal tissues (p < 0.001, Table 1, Fig. 1D). Our results indicate that the expression levels of PFKFB family members differs between patients with and without oral cancer.

**Table 1:** The comparison of gene expressions of PFKFB family members between tumor adjacent normal and tumor tissues in oral cancer patients from TCGA database

Variables	Tumor adjacent normal (n = 30)		Tumor (n = 315)		*p*-value^*^
	Mean ± SD	Median	Mean ± SD	Median	
PFKFB1	3.54 ± 2.11	2.7663	2.03 ± 0.91	1.9923	0.001
PFKFB2	9.51 ± 0.49	9.3475	9.41 ± 0.75	9.4500	0.322
PFKFB3	10.82 ± 0.89	10.8854	11.08 ± 0.82	11.0748	0.098
PFKFB4	7.48 ± 0.85	7.5412	8.21 ± 0.96	8.0848	< 0.001

*SD* standard deviation

^*^p values were estimated by student’s t- test

### Association between the expression of PFKFB family members and the prognosis of oral cancer patients

**Fig. 2:**
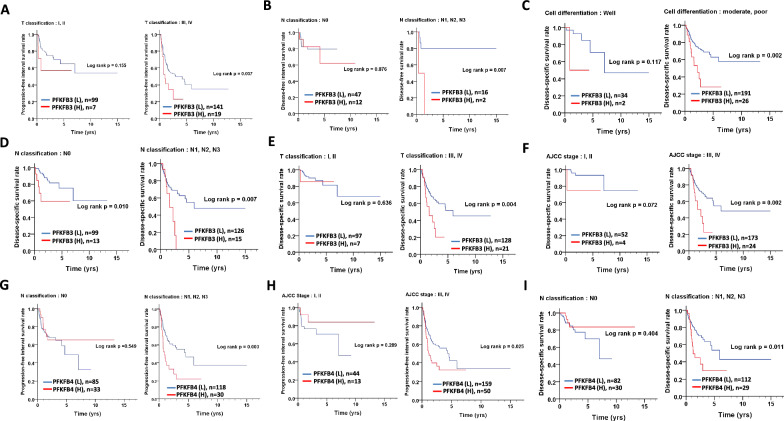
Different prognostic roles of oral cancer patients stratified by different clinicopathological outcomes depending on levels of *PFKFB3* and *PFKFB4*. **A**–**F** The association of high and low levels of *PFKFB3* with PFI, DFI, DSS in oral cancer patients stratified by cell differentiation, N-classification, T-classification, and pathological stage. **G**–**I** The association of high and low levels of *PFKFB4* with PFI and DSS in oral cancer patients stratified by pathological stage and N-classification

High expression of *PFKFBs* were associated with prognosis in many cancer patients [8]. However, their prognostic roles in oral cancer patients are still unknown. Next, we analyzed the association between the expression of PFKFB family members and various measures of survival, namely OS, PFI, DFI, and DSS. As data shown, high *PFKFB3* expression was associated with poor OS [crude hazard ratio [25] = 2.77 (1.02–7.51), p = 0.046, Table 2; p = 0.0037, Fig. 1E], poor PFI [adjusted hazard ratio (AHR) = 1.85 (1.08–3.19), p = 0.025, Table 2; p = 0.004, Fig. 1F]. Moreover, high PFKFB3 expression was not related to DFI [1.82 (0.64–5.21), p = 0.264, Table 2; p = 0.257, Fig. 1G] but to DSS [AHR = 2.43 (1.36–4.37), p = 0.003, Table 2; p < 0.001, Fig. 1H] in patients with oral cancer. *PFKFB1* and *PFKFB2* expression was not associated with OS, PFI, DFI, or DSS in patients with oral cancer when their data were stratified by clinicopathological outcome (Table 2, Additional file 1: Table S1 and Table S2). However, high *PFKFB3* expression was associated with a short PFI in patients with larger tumor size (T classification, III and IV, AHR = 1.86, p = 0.046, Table 3; p = 0.037, Fig. 2A]. High *PFKFB3* expression was associated with a short DFI in patients with lymph node metastasis [N classification, N1, N2 and N3, AHR = 7.88, p = 0.042, Table 3; p = 0.007, Fig. 2B]. High *PFKFB3* expression was associated with poor DSS in patients with moderate or poor cell differentiation [AHR = 2.22, p = 0.009, Table 3; p = 0.002, Fig. 2C], lymph node metastasis [N1, N2, and N3, AHR = 2.12, p = 0.04, Table 3; p = 0.007, Fig. 2D], larger tumors [T3 and T4, AHR = 2.38, p = 0.006, Table 3; p = 0.004, Fig. 2E] and advanced pathological stages [III or IV; AHR = 2.35, p = 0.006, Table 3; p = 0.002, Fig. 2F],

**Table 2:** The association of gene expression of PFKFB family members with survival in oral cancer patients from TCGA database

Variable	ROC	No. (%)	CHR (95% CI)	p value^*^	AHR (95% CI)	*p value*^†^
Overall survival						
PFKFB1	Low	265 (99.6)	1		1	
	High	1 (0.4)	5.33 (0.73–38.66)	0.098	4.25 (0.59–30.89)	0.153
PFKFB2	Low	210 (78.9)	1		1	
	High	56 (21.1)	1.20 (0.77–1.86)	0.421	1.28 (0.82–2.00)	0.274
PFKFB3	Low	25 (9.4)	1		1	
	High	241 (90.6)	2.77 (1.02–7.51)	0.046	1.93 (0.70–5.31)	0.201
PFKFB4	Low	164 (61.7)	1		1	
	High	102 (38.3)	1.21 (0.83–1.75)	0.323	1.13 (0.78–1.64)	0.519
Progression-free interval survival						
PFKFB1	Low	71 (26.7)	1		1	
	High	195 (73.3)	1.18 (0.75–1.84)	0.480	1.22 (0.78–1.92)	0.381
PFKFB2	Low	243 (91.4)	1		1	
	High	23 (8.6)	1.15 (0.60–2.20)	0.680	1.32 (0.67–2.59)	0.418
PFKFB3	Low	240 (90.2)	1		1	
	High	26 (9.8)	2.14 (1.25–3.65)	0.005	1.85 (1.08–3.19)	0.025
PFKFB4	Low	203 (76.3)	1		1	
	High	63 (23.7)	1.35 (0.88–2.05)	0.166	1.38 (0.91–2.11)	0.133
Disease-free interval survival						
PFKFB1	Low	241 (95.3)	1		1	
	High	12 (4.7)	2.20 (0.50–9.67)	0.295	2.24 (0.51–9.84)	0.286
PFKFB2	Low	201 (79.4)	1		1	
	High	52 (20.6)	21.62 (0.00–673172.18)	0.560	535,827.78 (0.00-)	0.987
PFKFB3	Low	225 (88.9)	1		1	
	High	28 (11.1)	1.82 (0.64–5.21)	0.264	1.92 (0.66–5.56)	0.229
PFKFB4	Low	194 (76.7)	1		1	
	High	59 (23.3)	1.61 (0.59–4.36)	0.350	1.51 (0.55–4.15)	0.419
Disease-specific survival						
PFKFB1	Low	16 (20.8)	1		1	
	High	61 (79.2)	1.58 (0.64–3.93)	0.325	1.38 (0.55–3.44)	0.490
PFKFB2	Low	3 (3.9)	1		1	
	High	74 (96.1)	1.45 (0.84–2.50)	0.188	1.51 (0.87–2.63)	0.146
PFKFB3	Low	63 (81.8)	1		1	
	High	14 (18.2)	2.74 (1.54–4.87)	0.001	2.43 (1.36–4.37)	0.003
PFKFB4	Low	35 (45.5)	1		1	
	High	42 (54.5)	1.35 (0.80–2.28)	0.259	1.43 (0.85–2.41)	0.184

*CI* confidence interval, *CHR* crude hazard ratio, *AHR* adjusted hazard ratio ^†^p values were adjusted for cell differentiation (moderate + poor vs. well) and AJCC pathological stage (stage III + IV vs stageI + II) by multivariate Cox’s regression

^*^p values were estimated by Cox’s regression

**Table 3:** The association of PFKFB3 expression with prognosis in oral cancer patients stratified by different clinicopathological features

PFKFB3	ROC	OS			PFI			DFI			DSS		
		No	AHR	*p*	No	AHR	*p*	No	AHR	*p*	No	AHR	*p*
Sex													
Female	Low	9	1		74	1		25	1		69	1	
	High	74	1.48	0.707^a^	9	2.12	0.149^a^	8	2.09	0.377^a^	9	2.65	0.145^a^
Male	Low	16	1		166	1		38	1		156	1	
	High	167	2.01	0.240^a^	17	1.78	0.079^a^	6	1.81	0.457^a^	19	2.40	0.009^a^
Age													
< = 60	Low	9	1		105	1		20	1		101	1	
	High	104	2.27	0.423^a^	8	1.42	0.468^a^	5	0.00	0.979^a^	9	1.85	0.211^a^
> 60	Low	16	1		135	1		43	1		124	1	
	High	137	1.85	0.307^a^	18	2.14	0.026^a^	9	3.13	0.049^a^	19	2.76	0.008^a^
Cell differentiation													
Well	Low	6	1		37	1		14	1		34	1	
	High	32	558328.67	0.980^b^	1	29.22	0.017^b^	4	1.09	0.950^b^	2	10.79	0.062^b^
Moderate + Poor	Low	19	1		203	1		49	1		191	1	
	High	209	1.46	0.463^b^	25	1.71	0.060^b^	10	2.27	0.178^b^	26	2.22	0.009^b^
N classification													
N0	Low	13	1		107	1		47	1		99	1	
	High	105	1.45	0.624^e^	11	2.01	0.130^e^	12	1.10	0.896^e^	13	2.72	0.060^e^
N1,N2,N3	Low	12	1		133	1		16	1		126	1	
	High	136	1.96	0.352^e^	15	1.76	0.107^e^	2	7.88	0.042^e^	15	2.12	0.040^e^
T classification													
T1 + T2	Low	17	1		99	1		52	1		97	1	
	High	89	2.16	0.300^d^	7	1.89	0.303^d^	8	1.66	0.522^d^	7	1.66	0.631^d^
T3 + T4	Low	8	1		141	1		11	1		128	1	
	High	152	1.33	0.696^d^	19	1.86	0.046^d^	6	7.82	0.114^d^	21	2.38	0.006^d^
AJCC pathological stage													
I + II	Low	10	1		54	1		39	1		52	1	
	High	47	2.32	0.425	3	6.31	0.019^c^	7	0.88	0.909^c^	4	6.86	0.105^c^
III + VI	Low	15	1		186	1		24	1		173	1	
	High	194	1.80	0.317^c^	23	1.72	0.068^c^	7	3.49	0.077^c^	24	2.35	0.006^c^

*OS* overall survival, *PFI* progression-free interval survival, *DFI* disease-specific survival, *DSS* disease-specific survival, *ROC* receiver operating characteristic curve, *AJCC* American Joint Committee on Cancer, *CI* confidence interval, *AHR* adjusted hazard ratio

^a^Adjusted for cell differentiation (moderate + poor vs. well) and AJCC pathological stage (stage III + IV vs stage I + II)

^b^Adjusted for AJCC pathological stage (stage III + IV vs stage I + II)

^c^Adjusted for cell differentiation (moderate + poor vs. well)

^d^Adjusted for cell differentiation (moderate + poor vs. well) and N classification (N1, N2 vs N0)

^e^Adjusted for cell differentiation (moderate + poor vs. well) and T classification (T3, T4 vs T1 + T2)

Moreover, high *PFKFB4* expression was associated with a short PFI in patients with lymph node metastasis [N1, N2, and N3, AHR = 2.00, p = 0.009, Table 4; p = 0.003, Fig. 2G] and late pathological stages [III and IV; AHR = 1.63, p = 0.03, Table 4; p = 0.025, Fig. 2H]. High *PFKFB4* expression was also associated with poor DSS in patients with lymph node metastasis [N1, N2, and N3, AHR = 1.88, p = 0.041, Table 4; p = 0.011, Fig. 2I]. Our results indicate that *PFKFB3* and *PFKFB4* expression levels have different effects on the prognosis of oral cancer patients with different clinicopathological outcomes.

**Table 4:** The association of PFKFB4 expression with prognosis in oral cancer patients stratified by different clinicopathological features

PFKFB4	ROC	OS				PFI			DFI			DSS		
		No	AHR		*p*	No	AHR	*p*	No	AHR	*p*	No	AHR	*p*
Sex														
Female	Low	54	1			66	1		16	1		63	1	
	High	29	0.48		0.063^a^	17	0.74	0.521^a^	17	0.66	0.624^a^	15	0.58	0.409^a^
Male	Low	110	1			137	1		19	1		131	1	
	High	73	1.50		0.076^a^	46	1.80	0.018^a^	25	3.30	0.136^a^	44	1.77	0.057^a^
Age														
< = 60	Low	70	1			87	1		10	1		84	1	
	High	43	1.12		0.713^a^	26	1.45	0.266^a^	15	494718.82	0.973^a^	26	1.10	0.823^a^
> 60	Low	94	1			116	1		25	1		110	1	
	High	59	1.08		0.770^a^	37	1.29	0.379^a^	27	1.05	0.931^a^	33	1.67	0.150^a^
Cell differentiation														
Well	Low	25	1			30	1		8	1		30	1	
	High	13	1.86		0.292^b^	8	0.83	0.812^b^	10	3.77	0.355^b^	6	1.25	0.848^b^
Moderate + Poor	Low	139	1			173	1		27	1		164	1	
	High	89	1.04		0.864^b^	55	1.42	0.114^b^	32	1.44	0.522^b^	53	1.45	0.180^b^
N classification														
N0	Low	67	1			85	1		26	1		82	1	
	High	51	0.84		0.604^e^	33	0.75	0.445^e^	33	0.99	0.992^e^	30	0.61	0.374^e^
N1,N2,N3	Low	97	1			118	1		9	1		112	1	
	High	51	1.20		0.459^e^	30	2.00	0.009^e^	9	3.48	0.309^e^	29	1.88	0.041^e^
T classification														
T1 + T2	Low	73	1			85	1		34	1		83	1	
	High	33	1.88		0.125^d^	21	1.26	0.604^d^	26	1.75	0.344^d^	21	2.18	0.220^d^
T3 + T4	Low	91	1			118	1		1	1		111	1	
	High	69	0.91		0.678^d^	42	1.41	0.173^d^	16	0.29	0.458^d^	38	1.26	0.438^d^
AJCC pathological stage														
I + II	Low	39	1			44	1		25	1		43	1	
	High	18	0.73		0.649^c^	13	0.45	0.292^c^	21	1.34	0.667^c^	13	0.00	0.973^c^
III + VI	Low	125	1			159	1		10	1		151	1	
	High	84	1.16		0.460^c^	50	1.63	0.030^c^	21	2.28	0.339^c^	46	1.65	0.066^c^

*OS* overall survival, *PFI* progression-free interval survival, *DFI* disease-specific survival, *DSS* disease-specific survival, *ROC* receiver operating characteristic curve, *AJCC* American Joint Committee on Cancer, *CI* confidence interval, *AHR* adjusted hazard ratio

^a^Adjusted for cell differentiation (moderate + poor vs. well) and AJCC pathological stage (stage III + IV vs stage I + II)

^b^Adjusted for AJCC pathological stage (stage III + IV vs stage I + II)

^c^Adjusted for cell differentiation (moderate + poor vs. well)

^d^Adjusted for cell differentiation (moderate + poor vs. well) and N classification (N1, N2 vs N0)

^e^Adjusted for cell differentiation (moderate + poor vs. well) and T classification (T3, T4 vs T1 + T2)

### Effects of PFKFB3 and PFKFB4 on the viability of OSCC cells

**Fig. 3:**
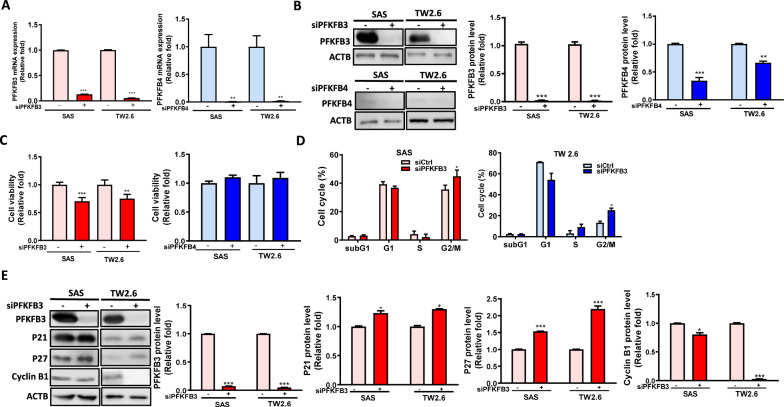
Cell viability and cell cycle progression of *PFKFB3*-or *PFKFB4*-silenced SAS and TW2.6 cells. The mRNA and protein levels of *PFKFB3* or *PFKFB4* were evaluated by **A** RT-qPCR and **B** Western blotting in *PFKFB3*- or *PFKFB4*-silenced cells. **C** Cell viability of *PFKFB3*-or *PFKFB4*-knockeddowned cells was analyzed by CellTiter-Glo assay. **D** Cell cycle progression of *PFKFB3*-silenced cells was analyzed by flow cytometry. **E** Cell cycle-related proteins in PFKFB3-silenced cells were analyzed by Western blotting. The 10 nM scrambled siRNA (siCtrl) or siRNA against *PFKFB3* or *PFKFB4* (si*PFKFB3* or si*PFKFB4*) were transfected into cells for 72 h. All data were represented as the average ± SD from 3 independent experiments. The significant differences between the scrambled control and knocked down cells were indicated as ^*^ p < 0.05, ^**^ p < 0.01, and ^***^ p < 0.001

*PFKFBs* play different roles in many cancers, such as cell viability and migration [8]. However, their biological roles in oral cancer are still unclear. To investigate if *PFKFB3 and PFKFB4* play role in cell viability of oral cancer cells, OSCC cells were knocked down using siRNA against *PFKFB3* or *PFKFB4*. After knockdown, the gene (Fig. 3A) and protein (Fig. 3B) levels of *PFKFB3* or *PFKFB4* were decreased. Moreover, the cell viability of *PFKFB3*-silenced OSCC cells was significantly lower, whereas the cell viability did not differ between scramble and *PFKFB4*-silenced OSCC cells (Fig. 3C). Also, the *PFKFB3*- or *PFKFB4*-silenced OSCC cells showed G2/M arrest (Fig. 3D), lower level of cell cycle regulator (cyclin B) but higher level of two cell cycle inhibitors (p21 and p27) (Fig. 3E). Moreover, high *PFKFB3* expression was associated with poor PFI and DSS in OSCC patients with larger tumors (Table 3). Our results indicate that *PFKFB3* (but not *PFKFB4*) might be involved in tumor growth through regulating G2/M cell cycle progression in OSCC.

### Effects of PFKFB3 and PFKFB4 on the migration of OSCC cells

**Fig. 4:**
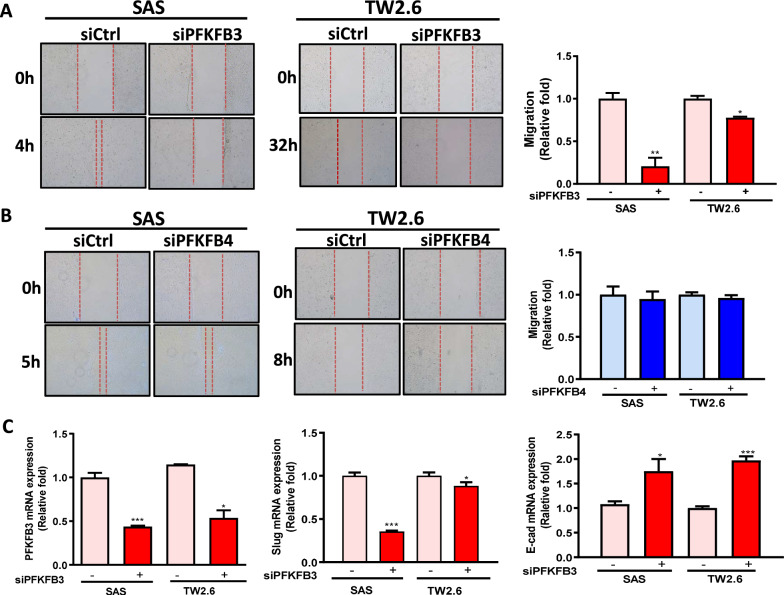
Cell migration and expression of EMT-related markers in *PFKFB3- or PFKFB4-silenced* SAS and TW2.6 cells. The cell migration of **A** *PFKFB3*-silenced and **B** *PFKFB4*-silenced cells was measured by the wound-healing assay. **C** Expression of EMT markers (Slug and E-cad) in *PFKFB3*-silenced cells were measured by qRT-PCR. The 10 nM scrambled siRNA (siCtrl) or siRNA against *PFKFB3 or PFKFB4 (siPFKFB3 or siPFKFB4)* were transfected into cells for 72 h. All data were represented as the average ± SD from 3 independent experiments. The significant differences between the scrambled control and knocked down cells were indicated as ^*^ p < 0.05, ^**^ p < 0.01, and ^***^ p < 0.001

We further investigated if PFKFB3 and PFKFB4 involve in migration of OSCC cells. SAS and TW2.6 cells were transiently knocked down with scramble siRNA and siRNA against *PFKFB3* or *PFKFB4*. The migration ability of *PFKFB3*-silenced cells was significantly weaker than that of the control cells (Fig. 4A). However, the migration ability of *PFKFB4*-silenced OSCC cells did not differ from that of the scramble cells (Fig. 4B). In addition, the expression of epithelial–mesenchymal transition (EMT) markers such as *Slug* was significantly lower, but that of *E-cadherin* was higher in the *PFKFB3*–knocked down OSCC cells (Fig. 4C). Moreover, high *PFKFB3* expression was associated with poor DFI and DSS in patients with OSCC with lymph node metastasis (Table 3). Our results indicate that *PFKFB3* might be involved in metastasis through EMT regulation in OSCC.

### Effects of PFKFB3 and PFKFB4 on the chemoresistance and cancer stemness of OSCC cells

**Fig. 5:**
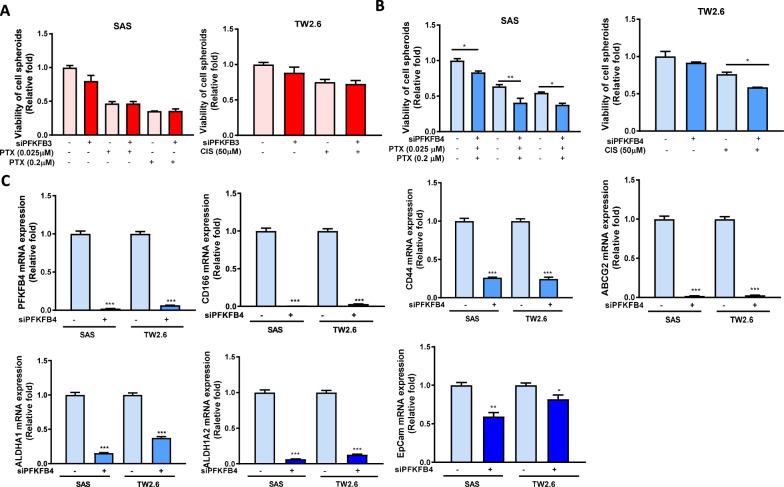
Sensitivity of cancer cell sphorids to cancer drugs in *PFKFB3*-or PFKFB4-silenced SAS and TW2.6 cells. The cell viability of spheroid cells silenced with scrambled siRNA or siRNA against **A** *PFKFB3* or **B** *PFKFB4* for 3 days then in the absence or presence of cisplatin (CIS, 50 µM) or paclitaxel (PTX, 0.025 and 2 µM) for 24 h was measured using the CellTiterGlo 3D assay. **C** mRNA levels of cancer stemness markers (*CD166, CD44, ABCG2, ALDH1A1, ALDH1A2* and *EpCAM*) in *PFKFB4*-silenced SAS cells were assessed by RT-qPCR. The 10 nM scrambled siRNA (siCtrl) or siRNA against *PFKFB3* or *PFKFB4* (si*PFKFB3* or si*PFKFB4*) were transfected into SAS cells for 72 h. All data were represented as the average ± SD from 3 independent experiments. The significant differences between scramble control and knocked down cells were indicated as ^*^ p < 0.05, ^**^ p < 0.01, and ^***^ p < 0.001

It is known that *PFKFBs* play roles in chemoresistance and cancer stemness [33]. We further explored the effects of *PFKFB3* and *PFKFB4* on chemoresistance and cancer stemness in OSCC cells. After knockdown, we observed no difference in viability of cell spheroids between *PFKFB3*-silenced SAS and TW2.6 cells untreated or treated with 0.025–0.2 μM of PTX or 50 μM of CIS (Fig. 5A). However, the *PFKFB4*-silenced SAS and TW2.6 cells exhibited significantly lower viability of cell spheroids in the presence of PTX and CIS compared with the scramble cells (Fig. 5B). To further confirm the role of PFKFB4 in cancer stemness-related chemoresistance, we investigated the expression of several cancer stemness markers, namely *CD44*, *CD166*, *ABCG2*, *ALDH1A1*, *ALDH1A2*, and *EpCAM* and found that their expressions were lower in *PFKFB4*–knocked down SAS and TW2.6 cells than scramble cells (Fig. 5C). Taken together, our results indicate that *PFKFB4* might be involved in the chemoresistance and cancer stemness of OSCC.

### The co-expressions of PFKFB3/cell cycle or EMT markers and PFKFB4/stemness markers in prognosis of oral cancer patients

**Fig. 6:**
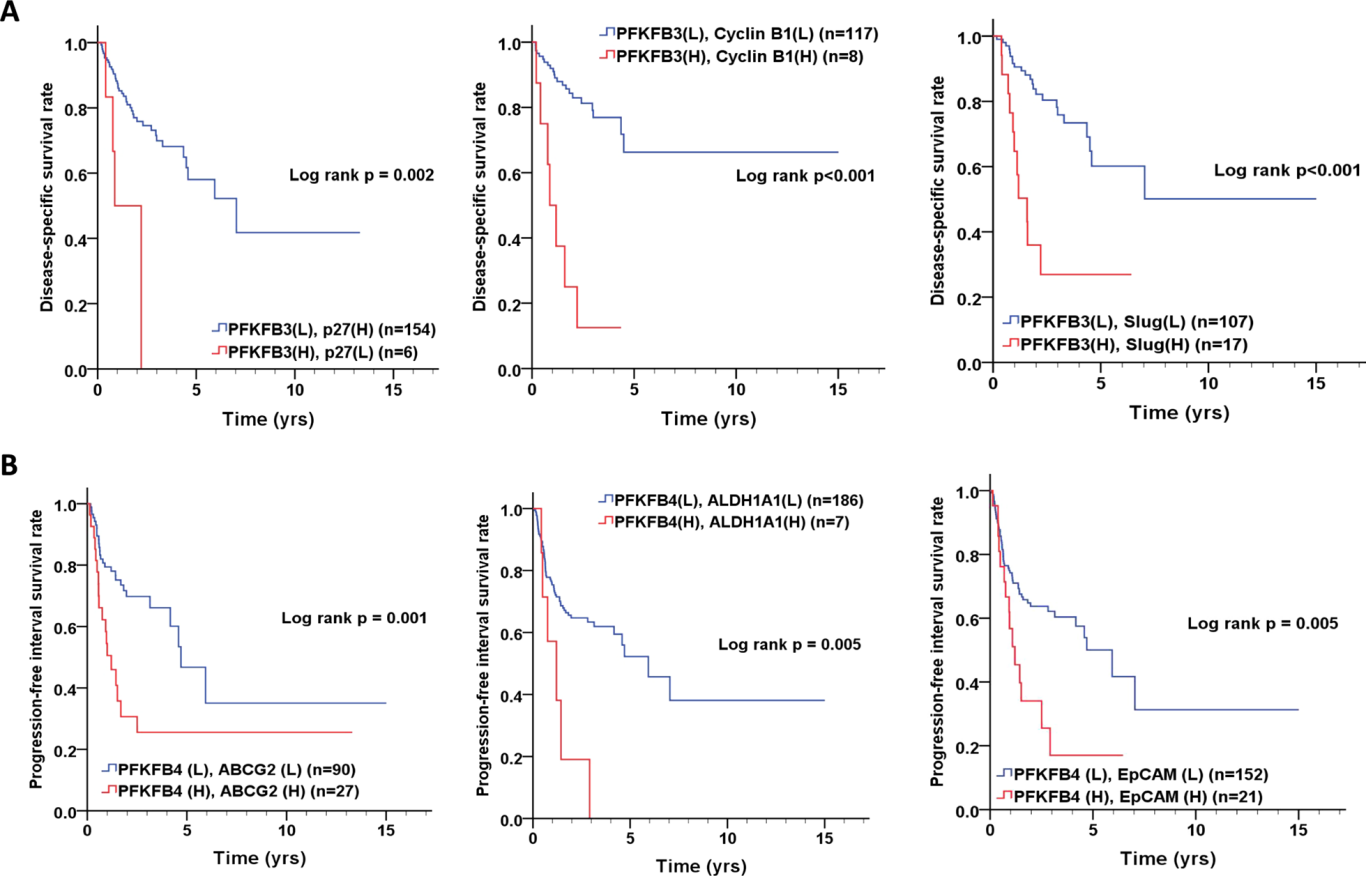
The association of co-expression of *PFKFB3/p27*, *PFKFB3/cyclin B1*, *PFKFB3/slug* and *PFKFB4*/stemness markers (*ABCG2, ALDH1A1* and *EpCAM*) with prognosis of oral cancer patients. **A** Co-expressions of *PFKFB3/p27*, *PFKFB3/cyclin B1*, *PFKFB3/slug* in DSS. **B** Co-expressions of *PFKFB4*/stemness markers (*ABCG2, ALDH1A1* and *EpCAM*) in PFI

Previous studies indicate that *PFKFBs* expression was significantly correlated with EMT-related [34] or stemness markers [33]. Moreover, our results indicated that PFKFB3 promoted tumor growth through regulating G2/M cell cycle and involved in metastasis through EMT regulation in OSCC cells. In oral cancer patients, we found that co-expressions of high PFKFB3/low p27 or high PFKFB3/high cyclin B1 or Slug were associated with poor DSS (Fig. 6A). Moreover, co-expressions of high PFKFB4/ high stemness markers such as *ABCG2, ALDH1A1* and *EpCAM* were also associated with poor PFI (Fig. 6B). Our analyzed data confirmed the possible effect of PFKFB3 in cell cycle progression and migration as well as  the effect of PFKFB4 in cancer stemness in OSCC.

## Discussion

PFKFBs are bidirectional glycolytic enzymes that control the steady-state cytoplasmic levels of F-2,6-BP, and increased F-2,6-BP concentration is a marker of glycolysis in many cancer cells [35]. PFKFBs have been reported to be involved in tumor progression and are considered potential biomarkers of various types of cancer [8]. However, their roles in oral cancer have not been reported. In the present study, we revealed that the expression of PFKFB4 was higher in the tumor tissues of oral cancer patients than in tumor-adjacent normal tissues. In addition, high *PFKFB3* expression was associated with a shorter survival  in oral cancer patients with poor cell differentiation, large tumors, and larger tumor sizes. High *PFKFB4* expression was associated with a shorter survival  in oral cancer patients with advanced lymph node metastasis and clinicopathological stages. Furthermore, *PFKFB3* is involved in the growth and metastasis of OSCC cells, but *PFKFB4* is involved in chemoresistance and the cancer stemness of OSCC cells. The co-expressions of PFKFB3/cell cycle or EMT markers and PFKFB4/stemness markers were associated with poor prognosis in oral cancer patients. These findings suggest that PFKFB family members have different biological roles and clinical significance in oral cancer patients.

*PFKFB1* was originally identified in tissues of the liver, muscle tissues, and fetal tissues but was not observed in cancer cells [36]. However, we discovered that *PFKFB1* had lower expression in tumor tissues than in normal tissues of patients with oral cancer. In addition, *PFKFB1* expression was not significantly associated with survival in patients with oral cancer. *PFKFB2* is expressed in the heart and kidney. *PFKFB2* is highly expressed in lung cancer [10], gastric cancer [11], melanoma [37], retinoblastoma [12], osteosarcoma [38], HCC [39] breast cancer [14], and prostate cancer [40]. Our results indicate that *PFKFB2* expression is not significantly different between tumor-adjacent normal and tumor tissues in oral cancer patients. In addition, *PFKFB2* expression was not associated with prognosis for oral cancer patients.

*PFKFB3* is frequently observed in pancreatic cancer, gastric cancer, nasopharyngeal carcinoma, and many other neoplasms [8]. *PFKFB3* is overexpressed in breast cancer [15], colon cancer [16], NSCLC [17], and HCC [18]. Moreover, high *PFKFB3* expression is linked to poor survival in brain tumors [9], indicating that *PFKFB3* might be a therapeutic target for various types of cancer. It is known that *PFKFB3* promotes the proliferation, invasion, and migration of breast cancer cells [41]. The blockage of *PFKFB3* decreases tumor growth and metastasis in head and neck squamous cell carcinoma (HNSCC) [42]. Our results indicate that *PFKFB3* was highly expressed in OSCC tissues and associated with a poor OS in oral cancer patients. We also discovered that the knockdown of *PFKFB3* significantly suppressed cell growth and migration of OSCC cells.

*PFKFB3*–knocked down HeLa cells have exhibited G1/S arrest [43]. *PFKFB3* expression has been induced during the G1/S transition [44]. The 3-(3-pyridinyl)-1-(4-pyridinyl)-2-propen-1-one (a kind of *PFKFB3* inhibitor) can induce G0/G1 arrest in A375 human melanoma cells [44] and G2/M arrest in Jurkat cells [45]. *PFKFB3* knockdown results in the G2/M arrest of HCC cells. *PFKFB3* upregulates some cyclin-dependent kinases (including Cdk-1, Cdc25C, and cyclin D3) and downregulates the p27 protein for G1/S transition and cell proliferation [46]. Our results indicated that PFKFB3 might control G2/M cell-cycle progression. On the other hand, *PFKFB3* modulates cell proliferation with the concomitant activation of NF-kB signaling in gastric cancer [47]. However, if *PFKFB3* is involved in cell growth through the regulation of NF-kB signaling in oral cancer, this topic will require further study.

Many studies have indicated that *PFKFB3* promotes metastasis by regulating EMT. *PFKFB3* knockdown inhibits invasiveness by upregulating E-cadherin and downregulating vimentin and N-cadherin levels in CNE2 human nasopharyngeal carcinoma cells [48]. The knockdown of *PFKFB3* reduces *Snail* expression and upregulates E-cadherin levels in pancreatic cancer cells [49]. Our results indicate that *PFKFB3* also regulates EMT-related *Slug* and *E-cad*, indicating that the upregulation of glycolysis promotes the EMT [50]. *PFKFB3*-modulating glycolysis is essential for lymphotoxin α–promoted tumor angiogenesis in HNSCC [51]. Many studies have indicated that *PFKFB3* is involved in the angiogenesis of OSCC [52], especially for lymphangiogenesis [53]. Our results suggest that oral cancer patients with higher *PFKFB3* expression exhibits lymph node metastasis (Table 4), implying that *PFKFB3* might promote lymphangiogenesis for lymph node metastasis.

Dysfunctional glycolysis results in drug resistance in clinical tumor therapy [27]. The knockdown of *PFKFB3* inhibits the expression of cancer stemness markers such as *CD133*, *ALDH1A1*, *CD44*, *Sox2*, and *ABCG2*, which are also associated with chemotherapy resistance [14]. Our results demonstrate that *PFKFB3* is involved in cell growth by regulating G2/M cell cycle progression and migration but not in chemoresistance and cancer stemness in OSCC cells.

We found that high *PFKFB3* expression was associated with a short PFI in patients with larger tumor size (T3 and T4) and was associated with a short DFI  in oral cancer patients with lymph node metastasis (N1, N2 and N3). In addition, high *PFKFB3* expression was also associated with poor DSS in oral cancer patients with moderate or poor cell differentiation, lymph node metastasis, and larger tumors.. Moreover, we found that *PFKFB3* is associated with cell growth and migration in OSCC cells. These results indicate that effects of *PFKFB3* on cell growth and metastasis are associated with tumor growth and metastasis in oral cancer patients.

Increasing *PFKFB4* expression contributes to the proliferation of HCC cells [54]. *PFKFB4* increases proliferative action in breast cancer cells [55]. PFKFB4 mediates *CD44*-driven proliferation in prostate cancer cells [56]. *PFKFB4* is key to the survival of glioma stem-like cells [57]. *PFKFB4* is involved in androgen-independent growth in human prostate cancer tissues [29]. It was reported that *PFKFB4* seems to contribute to tumor growth by regulating G1/0 phase progression for cell proliferation [8]. For example, the loss of *PFKFB4* induces cell cycle arrest in cervical cancer cells [58]. *PFKFB4* promotes G1/S transition for the cell proliferation of TNBC [20]. On the other hand, the knockdown of *PFKFB4* inhibits invasiveness through the upregulation of histone acetyltransferase GCN5 in IHH-4 thyroid cancer cells [59]. *PFKFB4* plays a role in the motility of cervical cancer cells [60]. *PFKFB4* activates cell migration in melanoma [24]. However, our study indicates that *PFKFB4* was not involved in cell growth and migration in OSCC cells.

*PFKFB4* enhances cancer stemness and contributes to chemoresistance to palbociclib in estrogen receptor–positive breast cancer [7]. *PFKFB4* is involved in chemoresistance to sunitinib in clear-cell renal cell carcinoma [23]. In addition, *PFKFB4* is involved in chemoresistance of HCC [18]. Previous studies showed that *CD44* may be a therapeutic target for glycolytic cancer cells that exhibit drug resistance [61]. Cancer cells with high glycolysis can release a large number of exosomes containing cancer stemness markers, including *ABCG2*, *ALDH1A1*, and *EpCAM* [62]. Our data also indicate that *PFKFB4* is involved in the chemoresistance of OSCC, which the inhibition of *PFKFB4* decreased the expression levels of *CD44*, *CD166*, *ABCG2*, *ALDH1A1*, *ALDH1A2*, and *EpCAM*. These data indicate that glucose metabolic reprogramming was involved in chemoresistance [62], which will need to be further verified.

We found that *PFKFB4* expression in tumor tissues was significantly higher than that in normal tissues and high *PFKFB4* expression was associated with a short PFI in oral cancer patients with lymph node metastasis and late pathological stages. High *PFKFB4* expression was also associated with poor DSS in oral cancer patients with lymph node metastasis. Moreover, we found that *PFKFB4* is associated with drug resistance and cancer stemness in OSCC cells. Since cancer stemness and drug resistance confer to survival of cancer patients, our results suggest that elevated *PFKFB4* might modulate signaling pathway required for drug resistance and cancer stemness, which in turn to contribute worse survival of oral cancer patients.

Our results showed that *PFKFB3* contributes to cellular proliferation and migration of OSCC. Previous study indicated that *PFKFB3* regulate both of proliferation and migration of ovarian cancer by regulating cytosolic protein tyrosine kinase 2 (focal adhesion kinase) [63]. Moreover, *PFKFB3* involves in the Ras signaling pathway, which is considered regulators of both proliferation and migration [64]. On the other hand, we revealed that *PFKFB4* involves in chemoresistance and cancer stemness of OSCC. It is reported that *PFKFB4*-mediated glycolysis pathway is associated with stemness features of breast cancer [65]. Moreover, *PFKFB4* modulates the chemoresistance of small-cell lung cancer by regulating autophagy [66]. According to above findings, *PFKFB3* and *PFKFB4* could contribute to many facets of oral cancer progression including controlling cell cycle progression, metastasis, and chemoresistance, which might not only act as regulators of glucose metabolism, but also act in a non-glycolysis-dependent manner (such as cell cycle regulation, autophagy, and transcriptional regulation) in OSCC [67]. Thus, the therapeutic implications of targeting *PFKFB3* and *PFKFB4* could disrupt glycolysis or Warburg effect and eliminate other signaling mechanisms for cancer progression.

Several studies revealed that targeting *PFKFB3* and *PFKFB4* could inhibit glycolysis in cancer cells [68]. Although *PFKFB3* inhibitors such as 3-(3-pyridinyl)-1-(4-pyridinyl)-2-propen-1-one (3PO) [69] or *PFKFB4* inhibitor such as 5-(n-(8-methoxy-4-quinolyl)amino)pentyl nitrate (5MPN) have been reported, their problems in low specificity and off targets are difficult to avoid [67]. Therefore, identifying more effective small molecule by computational approach involving virtual screening, drug-likeness, ADEM (absorption, distribution, metabolism, and excretion), molecular docking simulation, thermodynamic free energy calculations, per residue binding free energy contribution[70] and silico approach [71] or identifying plant extracts for inhibition of *PFKFB3* and *PFKFB4* enzymes for OSCC therapy is essential [72].

Our current study supports the clinical relevance and biological functions of *PFKFB3* and *PFKFB4* in oral cancer. Nevertheless, some of the detailed effects are still inconclusive due to the limitations of this study: (1) the cohort in TCGA database that we use to analyze the clinical significance of PFKFBs in most oral cancer patients is obtained from western countries, which needs more cohorts from other countries to further verify its importance in oral cancer; (2) the biological roles of PFKFBs was evaluated with oral cancer cell lines in this study, which needs the animal model to elucidate complex biological mechanisms that occur in OSCC patients; (3) The relation between *PFKFB3* and *PFKFB4* expression determine their prognostic value in several cancers [68], which needs further studies to analyze the relationship between both enzymes; (4) Previous study has shown that phosphorylation of PFKFB3 [73] and *PFKFB4* [74] isoenzyme increases their kinase activity. Thus, additional studies are needed to explore the protein levels of phosphorylated *PFKFB3* and *PFKFB4* in tumor tissues of OSCC patients.

## Conclusion

Our study first investigates roles of the PFKFB family in oral cancer patients. The overexpression of *PFKFB3* and *PFKFB4* was associated with low survival in oral cancer patients and was involved in cell growth/migration and chemoresistance/cancer stemness in OSCC cells, respectively. The co-expressions of PFKFB3/cell cycle or EMT markers and PFKFB4/stemness markers in oral cancer patients were also related to poor prognosis. Thus, we speculate that *PFKFB3* and *PFKFB4* might be potential prognostic biomarkers and therapeutic targets for OSCC patients.

### Supplementary Information

**Additional file 1: Table S1.**. The association of PFKFB1 expression with prognosis in oral cancer patients stratified by different clinicopathological features. **Table S2.**. The association of PFKFB2 expression with prognosis in oral cancer patients stratified by different clinicopathological features.

## Data Availability

The datasets used and/or analyzed during the current study are available from the corresponding author on reasonable request.

## Abbreviations

PFKFBs: Phosphofructokinase-fructose bisphosphatases
OSCC: Oral squamous cell carcinoma
NSCLC: Non-small cell lung cancer
HNSCC: Head and neck squamous cell carcinoma
HCC: Hepatocellular carcinoma
TNBC: Triple-negative breast cancer
DMEM: Dulbecco’s modified Eagle’s medium
OS: Overall survival
PFI: Progression-free interval survival
DSS: Disease-specific survival
DFI: Disease-free interval survival
CHR: Crude hazard ratio
AHR: Adjusted hazard ratio
EMT: Epithelial–mesenchymal transition
