# Supplementary material for: Comparative clinical significance and biological roles of PFKFB family members in oral squamous cell carcinoma
Source: Cancer Cell Int. 2023 Nov 2;23:257. doi: 10.1186/s12935-023-03110-6 (PMC10621127; doi:10.1186/s12935-023-03110-6)
Supplement: Supplementary file 1 — Additional file 1: Table S1.. The association of PFKFB1 expression with prognosis in oral cancer patients stratified by different clinicopathological features. Table S2.. The association of PFKFB2 expression with prognosis in oral cancer patients stratified by different clinicopathological features. [file 12935_2023_3110_MOESM1_ESM.docx]

Additional tables

| **Table S1.** The association of PFKFB1 expression with prognosis in oral cancer patients stratified by different  clinicopathological features. | | | | | | | | | | | | | |
| --- | --- | --- | --- | --- | --- | --- | --- | --- | --- | --- | --- | --- | --- |
| **PFKFB1** | **ROC** | **OS** | | | **PFI** | | | **DFI** | | | **DSS** | | |
|  |  | **No.** | AHR | *p* | **No.** | AHR | *p* | **No.** | AHR | *p* | **No.** | AHR | *p* |
| **Sex** | | | | | | | | | | | | | |
| Female | Low | 83 | 1 |  | 20 | 1 |  | 8 | 1 |  | 75 | 1 |  |
|  | High | 0 | IN |  | 63 | 1.32 | 0.575a | 25 | 2.91 | 0.368a | 3 | 0.00 | 0.976a |
| Male | Low | 182 | 1 |  | 51 | 1 |  | 8 | 1 |  | 166 | 1 |  |
|  | High | 1 | 3.93 | 0.178a | 132 | 1.20 | 0.496a | 36 | 2.66 | 0.355a | 9 | 1.98 | 0.149a |
| **Age** | | | | | | | | | | | | | |
| <=60 | Low | 112 | 1 |  | 32 | 1 |  | 5 | 1 |  | 106 | 1 |  |
|  | High | 1 | 6.69 | 0.068a | 81 | 1.72 | 0.135a | 20 | 0.93 | 0.951a | 4 | 9.96 | 0.002a |
| >60 | Low | 153 | 1 |  | 39 | 1 |  | 11 | 1 |  | 135 | 1 |  |
|  | High | 0 | IN |  | 114 | 0.93 | 0.807a | 41 | 4.07 | 0.185a | 8 | 0.63 | 0.526a |
| **Cell differentiation** | | | | | | | | | | | | | |
| Well | Low | 38 | 1 |  | 8 | 1 |  | 4 | 1 |  | 34 | 1 |  |
|  | High | 0 | IN |  | 30 | 1.23 | 0.804b | 14 | 0.64 | 0.733b | 2 | 4.12 | 0.231b |
| Moderate  +Poor | Low | 227 | 1 |  | 63 | 1 |  | 12 | 1 |  | 207 | 1 |  |
|  | High | 1 | 4.46 | 0.140b | 165 | 1.25 | 0.362b | 47 | 3.66 | 0.212b | 10 | 1.21 | 0.720b |
| **N classification** | | | | | | | | | | | | | |
| N0 | Low | 118 | 1 |  | 29 | 1 |  | 12 | 1 |  | 109 | 1 |  |
|  | High | 0 | IN |  | 89 | 1.67 | 0.219e | 47 | 1.52 | 0.595e | 3 | 5.89 | 0.021e |
| N1,N2,N3 | Low | 147 | 1 |  | 42 | 1 |  | 4 | 1 |  | 132 | 1 |  |
|  | High | 1 | 7.65 | 0.052e | 106 | 1.05 | 0.863e | 14 | 345490.87 | 0.981e | 9 | 1.07 | 0.912e |
| **T classification** | | | | | | | | | | | | | |
| T1+T2 | Low | 105 | 1 |  | 25 | 1 |  | 13 | 1 |  | 98 | 1 |  |
|  | High | 1 | 6.84 | 0.071d | 81 | 1.00 | 0.990d | 47 | 3.18 | 0.269d | 6 | 1.16 | 0.889d |
| T3+T4 | Low | 160 | 1 |  | 46 | 1 |  | 3 | 1 |  | 143 | 1 |  |
|  | High |  | IN |  | 114 | 1.30 | 0.337d | 14 | 0.89 | 0.933d | 6 | 1.67 | 0.330d |
| **AJCC pathological stage** | | | | | | | | | | | | | |
| I+II | Low | 57 | 1 |  | 13 | 1 |  | 9 | 1 |  | 55 | 1 |  |
|  | High | 0 | IN |  | 44 | 1.06 | 0.932c | 37 | 2.13 | 0.476c | 1 | 0.00 | 0.993c |
| III+VI | Low | 208 | 1 |  | 58 | 1 |  | 7 | 1 |  | 186 | 1 |  |
|  | High | 1 | 4.18 | 0.158e | 151 | 1.23 | 0.397c | 24 | 2.60 | 0.379c | 11 | 1.38 | 0.493c |
| *Abbreviations: OS, overall survival; PFI, progression-free interval survival; DFI, disease-specific survival; DSS, disease-specific survival; ROC, receiver operating characteristic curve; AJCC, American Joint Committee on Cancer; CI, confidence interval; AHR, adjusted hazard ratio*  *^a^Adjusted for cell differentiation (moderate+poor vs. well) and AJCC pathological stage (stage III+ IV vs stage I+II).*  *^b^Adjusted for AJCC pathological stage (stage III+ IV vs stage I+II).*  *^c^Adjusted for cell differentiation (moderate+poor vs. well).*  *^d^Adjusted for cell differentiation (moderate+poor vs. well) and N classification (N1, N2 vs N0). ^e^Adjusted for cell differentiation (moderate+poor vs. well) and T classification (T3, T4 vs T1+T2). IN: incalculable* | | | | | | | | | | | | | |

| **Table S2.** The association of PFKFB2 expression with prognosis in oral cancer patients stratified by different  clinicopathological features. | | | | | | | | | | | | | |
| --- | --- | --- | --- | --- | --- | --- | --- | --- | --- | --- | --- | --- | --- |
| **PFKFB2** | **ROC** | **OS** | | | **PFI** | | | **DFI** | | | **DSS** | | |
|  |  | **No.** | AHR | *p* | **No.** | AHR | *p* | **No.** | AHR | *p* | **No.** | AHR | *p* |
| **Sex** | | | | | | | | | | | | | |
| Female | Low | 66 | 1 |  | 79 | 1 |  | 1 | 1 |  | 62 | 1 |  |
|  | High | 17 | 0.42 | 0.098a | 4 | 1.89 | 0.457a | 32 | 413916.27 | 0.993a | 16 | 0.88 | 0.842a |
| Male | Low | 144 | 1 |  | 164 | 1 |  | 2 | 1 |  | 139 | 1 |  |
|  | High | 39 | 1.74 | **0.035a** | 19 | 1.24 | 0.573a | 42 | 692173.31 | 0.989a | 36 | 1.74 | 0.089a |
| **Age** | | | | | | | | | | | | | |
| <=60 | Low | 89 | 1 |  | 102 | 1 |  | 1 | 1 |  | 86 | 1 |  |
|  | High | 24 | 2.36 | **0.016a** | 11 | 1.40 | 0.528a | 24 | 1040172.36 | 0.994a | 24 | 2.57 | **0.024a** |
| >60 | Low | 121 | 1 |  | 141 | 1 |  | 2 | 1 |  | 115 | 1 |  |
|  | High | 32 | 0.82 | 0.517a | 12 | 1.15 | 0.760a | 50 | 517568.90 | 0.988a | 28 | 1.03 | 0.941a |
| **Cell differentiation** | | | | | | | | | | | | | |
| Well | Low | 25 | 1 |  | 31 | 1 |  | 0 | 1 |  | 25 | 1 |  |
|  | High | 13 | 1.45 | 0.525b | 7 | 1.05 | 0.944b | 18 | incalculable |  | 11 | 1.15 | 0.871b |
| Moderate  +Poor | Low | 185 | 1 |  | 212 | 1 |  | 3 | 1 |  | 176 | 1 |  |
|  | High | 43 | 1.20 | 0.475b | 16 | 1.33 | 0.471b | 56 | 486758.74 | 0.986b | 41 | 1.55 | 0.141b |
| **N classification** | | | | | | | | | | | | | |
| N0 | Low | 93 | 1 |  | 109 | 1 |  | 2 | 1 |  | 91 | 1 |  |
|  | High | 25 | 0.98 | 0.955e | 9 | 0.78 | 0.714e | 57 | 177033.82 | 0.984e | 21 | 0.77 | 0.681e |
| N1,N2,N3 | Low | 117 | 1 |  | 134 | 1 |  | 1 | 1 |  | 110 | 1 |  |
|  | High | 31 | 1.36 | 0.272e | 14 | 1.80 | 0.150e | 17 | 278562.36 | 0.989e | 31 | 1.74 | 0.085e |
| **T classification** | | | | | | | | | | | | | |
| T1+T2 | Low | 86 | 1 |  | 97 | 1 |  | 3 | 1 |  | 85 | 1 |  |
|  | High | 20 | 1.58 | 0.328d | 9 | 0.41 | 0.385d | 57 | 614679.26 | 0.987d | 19 | 0.49 | 0.490d |
| T3+T4 | Low | 124 | 1 |  | 146 | 1 |  | 0 | 1 |  | 116 | 1 |  |
|  | High | 36 | 1.17 | 0.550d | 14 | 1.84 | 0.117d | 17 | incalculable |  | 33 | 1.68 | 0.087d |
| **AJCC pathological stage** | | | | | | | | | | | | | |
| I+II | Low | 47 | 1 |  | 54 | 1 |  | 2 | 1 |  | 47 | 1 |  |
|  | High | 10 | 0.95 | 0.952 | 3 | 0.00 | 0.984c | 44 | 582765.30 | 0.990c | 9 | 0.00 | 0.986c |
| III+VI | Low | 163 | 1 |  | 189 | 1 |  | 1 | 1 |  | 154 | 1 |  |
|  | High | 46 | 1.31 | 0.264c | 20 | 1.56 | 0.204c | 30 | 506761.22 | 0.992c | 43 | 1.64 | 0.084c |
| *Abbreviations: OS, overall survival; PFI, progression-free interval survival; DFI, disease-specific survival ; DSS, disease-specific survival ; ROC, receiver operating characteristic curve; AJCC, American Joint Committee on Cancer; CI, confidence interval; AHR, adjusted hazard ratio*  *^a^Adjusted for cell differentiation (moderate+poor vs. well) and AJCC pathological stage (stage III+ IV vs stage I+II).*  *^b^Adjusted for AJCC pathological stage (stage III+ IV vs stage I+II).*  *^c^Adjusted for cell differentiation (moderate+poor vs. well).*  *^d^Adjusted for cell differentiation (moderate+poor vs. well) and N classification (N1, N2 vs N0).*  *^e^Adjusted for cell differentiation (moderate+poor vs. well) and T classification (T3, T4 vs T1+T2).* | | | | | | | | | | | | | |
